# Gene therapy for urea cycle defects: An update from historical perspectives to future prospects

**DOI:** 10.1002/jimd.12609

**Published:** 2023-04-18

**Authors:** Claire Duff, Ian E. Alexander, Julien Baruteau

**Affiliations:** ^1^ Genetics and Genomic Medicine Department, Great Ormond Street Institute of Child Health University College London London UK; ^2^ Gene Therapy Research Unit, Children's Medical Research Institute, Faculty of Medicine and Health The University of Sydney and Sydney Children's Hospitals Network Westmead New South Wales Australia; ^3^ Discipline of Child and Adolescent Health The University of Sydney Westmead New South Wales Australia; ^4^ National Institute of Health Research Great Ormond Street Biomedical Research Centre London UK; ^5^ Metabolic Medicine Department Great Ormond Street Hospital for Children NHS Foundation Trust London UK

**Keywords:** ammonia, argininaemia, argininosuccinic aciduria, citrullinaemia, ornithine transcarbamylase, urea cycle, urea cycle defect

## Abstract

Urea cycle defects (UCDs) are severe inherited metabolic diseases with high unmet needs which present a permanent risk of hyperammonaemic decompensation and subsequent acute death or neurological sequelae, when treated with conventional dietetic and medical therapies. Liver transplantation is currently the only curative option, but has the potential to be supplanted by highly effective gene therapy interventions without the attendant need for life‐long immunosuppression or limitations imposed by donor liver supply. Over the last three decades, pioneering genetic technologies have been explored to circumvent the consequences of UCDs, improve quality of life and long‐term outcomes: adenoviral vectors, adeno‐associated viral vectors, gene editing, genome integration and non‐viral technology with messenger RNA. In this review, we present a summarised view of this historical path, which includes some seminal milestones of the gene therapy's epic. We provide an update about the state of the art of gene therapy technologies for UCDs and the current advantages and pitfalls driving future directions for research and development.

## 1 INTRODUCTION

Urea cycle defects (UCDs) are severe inherited metabolic diseases (IMD), primarily involving the liver with high unmet needs. UCDs present with an overall incidence of 1 in 8200^1^ to 1 in 52 000^2^, ^3^, ^4^ live births. UCDs are characterised by acute life‐threatening hyperammonaemic decompensations from the neonatal period onwards, poor quality of life with a protein‐restricted diet and multiple daily medications, frequent medical reviews and hospital admissions, and a significant social impact.^5^

The urea cycle is a pathway which enables (i) the detoxification of neurotoxic ammonia, produced by protein catabolism, into urea and (ii) the synthesis of arginine, a precursor of multiple essential metabolites such as creatine, nitric oxide (NO), polyamines and agmatine. The liver is the only organ where all urea cycle enzymes are expressed, specifically in periportal hepatocytes, following well‐defined metabolic zonation of the hepatic lobule.^6^ Although the liver plays a key role in nitrogen wasting and ureagenesis, other organs express urea cycle‐related enzymes. The systemic endogenous arginine pool is mostly replenished by the kidney.^7^ The arginine‐NO pathway is essential for various key physiological roles such as enterocyte physiology,^8^, ^9^ angiogenesis,^10^ osteogenesis,^11^ neuronal redox status^12^ and cerebral motor control.^13^ The arginine–creatine pathway plays a role in neuronal and astrocytic differentiation.^14^

The urea cycle comprises six enzymes and two transporters. Three of the enzymes are mitochondrial; N‐acetylglutamate synthase, carbamoyl‐phosphate synthase I and ornithine transcarbamylase (OTC), and three are cytosolic; argininosuccinate synthase (ASS), argininosuccinate lyase (ASL) and arginase 1 (ARG1).^15^ In addition, the mitochondrial ornithine/citrulline antiporter enables the export of citrulline and the uptake of ornithine by mitochondria. Citrin is a hepatic mitochondrial carrier that transports aspartate from the mitochondria to the cytoplasm. UCDs are cell autonomous diseases with autosomal recessive inheritance except for X‐linked OTC deficiency (OMIM entries in the respective order of the text introduction of the proteins, #237310, #237300, #311250, #215700, #207900, #207800, #238970, #603859). OTC deficiency represents 55%–60% of all UCD patients, followed by ASL (15%–20%), ASS (10%–15%) and CPS1 (5%–10%) deficiencies.^2^, ^3^, ^4^

It is estimated that a limited increase in the residual activity of a deficient enzyme could significantly alleviate the severity of UCDs' presentation, by transforming a severe phenotype into a milder one with significantly reduced risk of hyperammonaemic decompensation.^12^, ^16^, ^17^, ^18^ While the threshold of enzymatic activity required to obtain complete phenotype correction remains unknown and is likely to be disease‐specific, it has been suggested to be in the order of 10%.^19^, ^20^ There are, however, a number of caveats built into this estimate and the gene transfer threshold in terms of the proportion of hepatocytes that must be successfully gene‐modified for robust therapeutic effect may well be higher. Irrespective, this low estimate is still significantly higher than the gene transfer efficiencies required for inherited liver diseases involving secreted proteins (non‐cell autonomous), such as haemophilia B whereas little a 2%–5% physiological levels of expression of factor IX, potentially from even fewer hepatocytes, is known to confer clinical benefit.^21^

**FIGURE 1:**
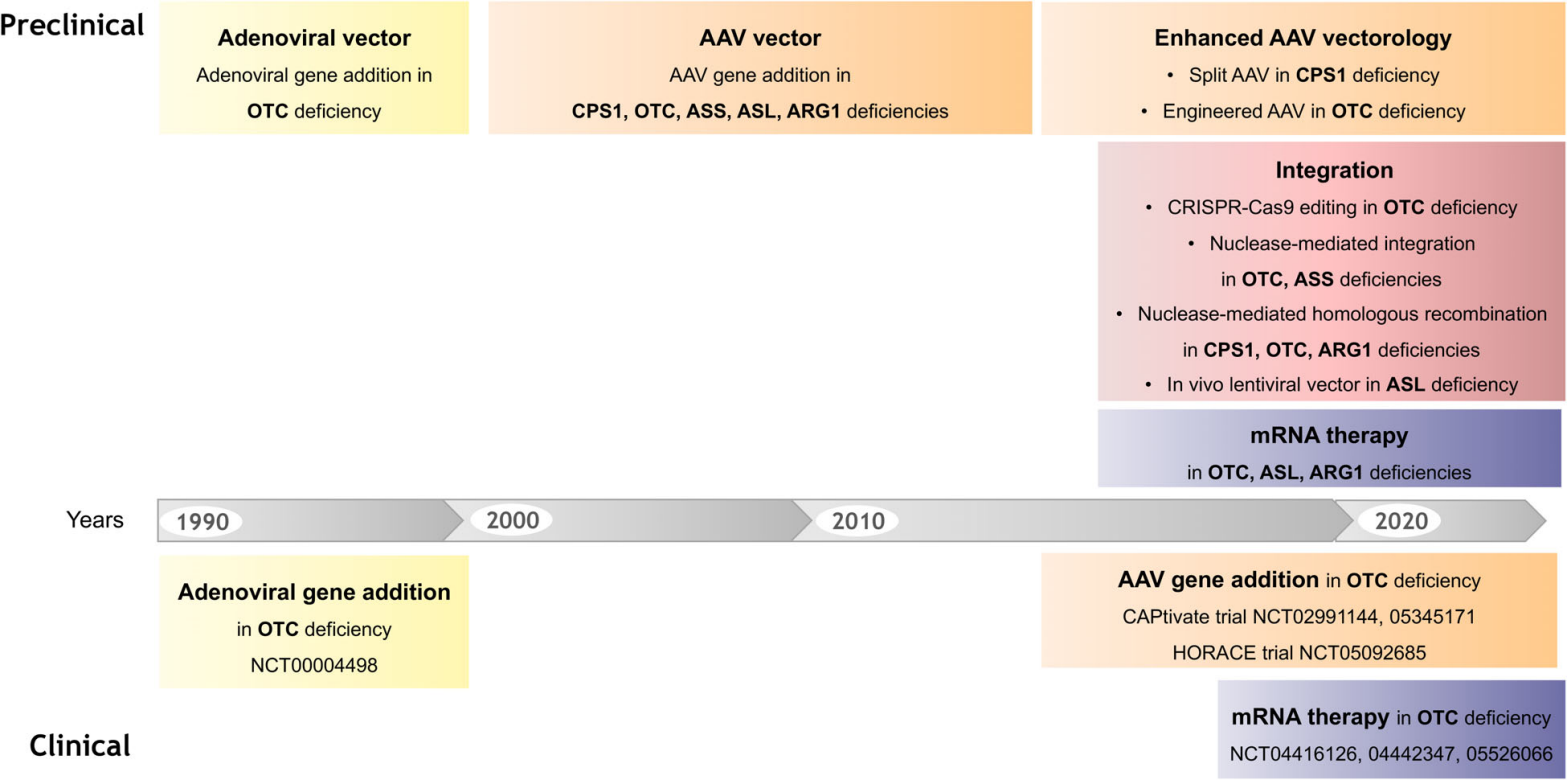
Preclinical and clinical development of gene therapy for urea cycle defects overtime. ARG1, arginase 1; ASL, argininosuccinate lyase; ASS, argininosuccinate synthase; CPSI, carbamoyl‐phosphate synthase I; OTC, ornithine transcarbamylase.

The gold standard and best‐accepted therapies for UCDs rely on a protein‐restricted diet, ammonia‐scavenging drugs and arginine/citrulline supplementation.^15^ This conventional approach enables patients to survive but has limitations as this does not eliminate the risk of acute hyperammonaemic decompensation. The only curative strategy is liver transplantation.^15^, ^22^ This is widely used in the USA on a prospective basis, that is, in infancy before risking hyperammonaemic decompensations in childhood.^23^, ^24^ In Europe, liver transplantation is often proposed in patients with unstable metabolic control and recurrent hyperammonaemias.^15^ Liver transplantation causes procedure‐related morbidity and requires lifelong immunosuppression. The limitations and morbidity associated with the current therapeutic options highlight the need for novel therapies with a more favourable interventional risk–benefit profile. This explains why UCDs have been targeted in multiple studies to develop proof‐of‐concept of novel gene therapy technologies over the last three to four decades (Figure 1). UCDs are also useful as models in which to explore and develop technological platforms for broader success in liver‐IMD.

## 2 DISEASE‐SPECIFIC LIMITATIONS

UCDs present with acute hyperammonaemia, which causes neurological sequelae and/or death if not rapidly treated. Some UCDs have phenotypic specificities with extra‐hepatic organ involvement and clinical manifestations that would not be adequately addressed by liver‐targeting gene therapy alone.

Occasionally UCD patients present with liver fibrosis^25^, ^26^, ^27^ and rarely cirrhosis.^28^, ^29^ The underlying pathophysiology is not well understood, and in the context of gene therapy, liver fibrosis is likely to reduce the efficiency of hepatocyte transduction and potentially impair biodistribution within the liver parenchyma.

ASL deficiency shows a systemic phenotype with high rates of liver complications (hepatitis, hepatomegaly, fibrosis, glycogen storage), neurological morbidity (developmental delay, epilepsy, movement disorder), arterial hypertension, chronic gastrointestinal and renal symptoms, hypertriglyceridaemia.^7^, ^30^ The pathophysiology is likely multifactorial, caused by toxicity of argininosuccinate, redox imbalance and consequences of arginine deficiency with NO, and polyamines and creatine deficiencies.^7^ ARG1 deficiency shows spastic diplegia and delayed myelination.^31^ Although liver‐targeting therapeutics, such as liver transplantation, can provide a systemic improvement of the underlying disease, it is unrealistic that a ‘liver only’ strategy could cure all extra‐hepatic manifestations.

## 3 UNDERSTANDING THE THERAPEUTIC CHALLENGE

A critical precondition for the development of successful gene therapy approaches is a deep understanding of target disease pathophysiology, the biology of the target cell population and the therapeutic reach of available gene transfer and genome editing technologies. UCD set a high bar, and the most compelling evidence of this comes from a lesson of nature. Heterozygous females with OTC deficiency, where one X chromosome carries the healthy OTC allele and the other X chromosome carries the mutant allele, have livers containing a mixed population of hepatocytes with normal or deficient/absent OTC enzymatic activity, depending on which X chromosome is inactivated in any given hepatocyte.^32^ The process of X chromosome inactivation occurs early in liver organogenesis when the number of hepatocyte progenitors is low and the stochastic nature of X chromosome inactivation can produce livers with significant skewing in X inactivation in favour of either the X chromosome carrying the wild‐type or mutant allele. Skewing in favour of the X chromosome carrying the wild‐type OTC allele reduces the proportion of hepatocytes expressing OTC and is associated with an increasingly severe phenotype. For example, heterozygous females with skewing down to 10% of hepatocytes expressing the wild‐type allele would be expected to have a severe disease phenotype. Given that male patients with 10% residual OTC activity would be expected to have less severe late‐onset disease,^33^ it becomes clear that the liver‐wide cellular biodistribution of OTC activity is critical. Normal physiological OTC activity in 10% of hepatocytes (90% with no activity) is not as effective for ureagenesis as liver‐wide sub‐physiological activity of 10%. Notably, female patients with a mixed population of OTC‐expressing hepatocytes, naturally model the post‐gene therapy liver, and support the conclusion that significantly more than 10% of hepatocytes will need to be genetically modified to confer robust therapeutic effect. Mathematical modelling of the urea cycle supports qualitatively similar conclusions for other UCDs.^34^

Achieving the gene transfer efficiencies required for therapeutic effects in UCDs is further complicated by the phenomenon of metabolic zonation, whereby multiple metabolic functions of the liver show distinct gradients of activity across the hepatic lobule between portal triads and central vein,^35^ with urea cycle enzymes most strongly expressed in hepatocytes located the periportal regions. As a consequence, gene therapy interventions for UCDs should ideally preferentially target this region of the hepatic lobule, as gene delivery/editing events near the central vein will contribute relatively little to the restoration of ureagenic activity. Early efforts to address this challenge are only just appearing in the gene transfer literature.^36^

Yet another important challenge, most notably in the paediatric context, is liver growth. This is a particularly important consideration when using episomal vector systems as hepatocellular replication results in dilution and loss of vector genomes over time with an associated decline in therapeutic effect. For this reason, there is a growing focus on technological approaches that result in permanent changes to the hepatocyte genome, whether by genomic integration of a therapeutic cassette or precise locus‐specific correction of the disease‐causing mutant locus using genome editing approaches.

Balancing these formidable challenges, the liver also has a number of properties that favour gene therapy interventions, particularly following systemic delivery by intravenous injection of gene transfer formulations, including those based on recombinant viruses and physicochemical approaches such as lipid nanoparticles. While the liver represents approximately 2%–3% of body weight it receives approximately 25% of cardiac output, representing very high relative blood flow.^37^ Thus, a high proportion of the initial gene transfer dose reaches the liver on first pass, where the second favourable feature of liver biology is encountered in the presence of a fenestrated vascular endothelium.^38^ This allows the gene transfer formulation in the intravascular compartment to make direct contact with the surface of hepatocytes without the need to penetrate an intact vascular endothelium.

A final favourable feature of the liver is its tolerogenic properties, most evident in the fact that livers can be successfully transplanted across HLA boundaries, unlike other solid organs subject to transplantation.^39^ This reduces the probability that transgenes expressed in the liver, even if they are effectively neo‐antigens, will induce an immune response through the presentation of transgene‐derived peptides via the HLA class I pathway. Notably, however, strong adaptive immune responses to structural elements of gene transfer vectors, such as the capsid of adeno‐associated virus vectors (rAAV), have been reported following liver‐targeted gene therapy trials in humans.^40^

## 4 1990S: FIRST STEPS ON AN ARDUOUS PATH: ADENOVIRAL VECTORS

The first clinical trial targeting UCDs was initiated in the late 1990s using adenoviral vectors for liver‐targeted gene expression. Adenoviruses are non‐enveloped double‐stranded DNA viruses. They accommodate a large 36 kb genome and transduce dividing and non‐dividing cells,^41^ but remain episomal and therefore have the potential to be lost from dividing cell populations over time. The most common serotype is serotype 5, which has high liver tropism. Adenoviruses are pro‐inflammatory and trigger sustained innate and adaptive immune responses.^42^ Despite attempts to reduce vector‐related immunity by deleting specific viral genomic sequences, various generations of adenoviral vectors have been developed. Proof of concept has been shown in OTC‐, ASS‐, ASL‐ and ARG1‐deficient murine models.^43^, ^44^, ^45^, ^46^, ^47^ A phase I/II gene therapy clinical trial recruited late‐onset OTC‐deficient adult patients to test the safety and efficacy of a second‐generation adenoviral 5 vector encoding the *hOTC* gene. One of the participants, Jesse Gelsinger, was dosed with the highest of the six escalating doses (6 × 10^11^ vg/kg) via the hepatic artery. He subsequently developed a severe immune response syndrome within 24 h and died of multi‐organ failure 4 days after adenoviral vector administration.^48^ Another subject received the same highest dose of adenoviral vector in this trial without lethal consequences. The cause of death was a severe capsid‐mediated innate immune response and cytokine storm. Preclinical studies had shown severe toxicity in monkeys at 17‐fold higher dose and minor laboratory abnormalities at the doses used in the study.^49^ This death sent a shock‐wave through the field of gene therapy, delivered a salutary lesson about risk–benefit in human trials and highlighted the urgent need to develop safer vectors.

## 5 2000S: PROGRESSING TOWARDS SAFER VIRAL VECTORS: THE AAV ERA

The early 2000s saw a focus on developing safer viral vectors, with intensifying interest in vectors derived from adeno‐associated virus (AAV). AAV is a single‐stranded DNA virus which is not associated with any known pathogenicity during acute seroconversion. Genomic integration is not an obligate element of the AAV viral life cycle, and the rate of integration of AAV vectors sequences is relatively low but still significant.^50^ Recent publications suggest a potential association between wild‐type AAV integration in the human liver and tumorigenicity,^51^, ^52^ however, the ontogeny of hepatocellular cancer is complex and multifactorial, rendering causality difficult to establish.^53^ Paediatric acute liver failure has also recently been reported with co‐helper viruses such as adeno‐ or helper viruses, but remains incompletely understood.^54^

Initial proof‐of‐concept studies using AAV‐mediated gene addition, performed with hepatotropic AAV serotypes such as AAV7, 8 or 9, have shown long‐term normalisation of the main biomarker, urinary orotate, in mild adult OTC‐deficient *Spf* ^*ash*^ mice.^55^ Moreover, the risk of long‐term liver genotoxicity appears to be low.^56^, ^57^ Noteworthy, however, is evidence that rare integration events in the Rian locus in mice treated in the newborn period can lead to hepatocellular carcinoma in aged mice,^58^ with the magnitude of the risk related to promoter–enhancer strength.^59^

**FIGURE 2:**
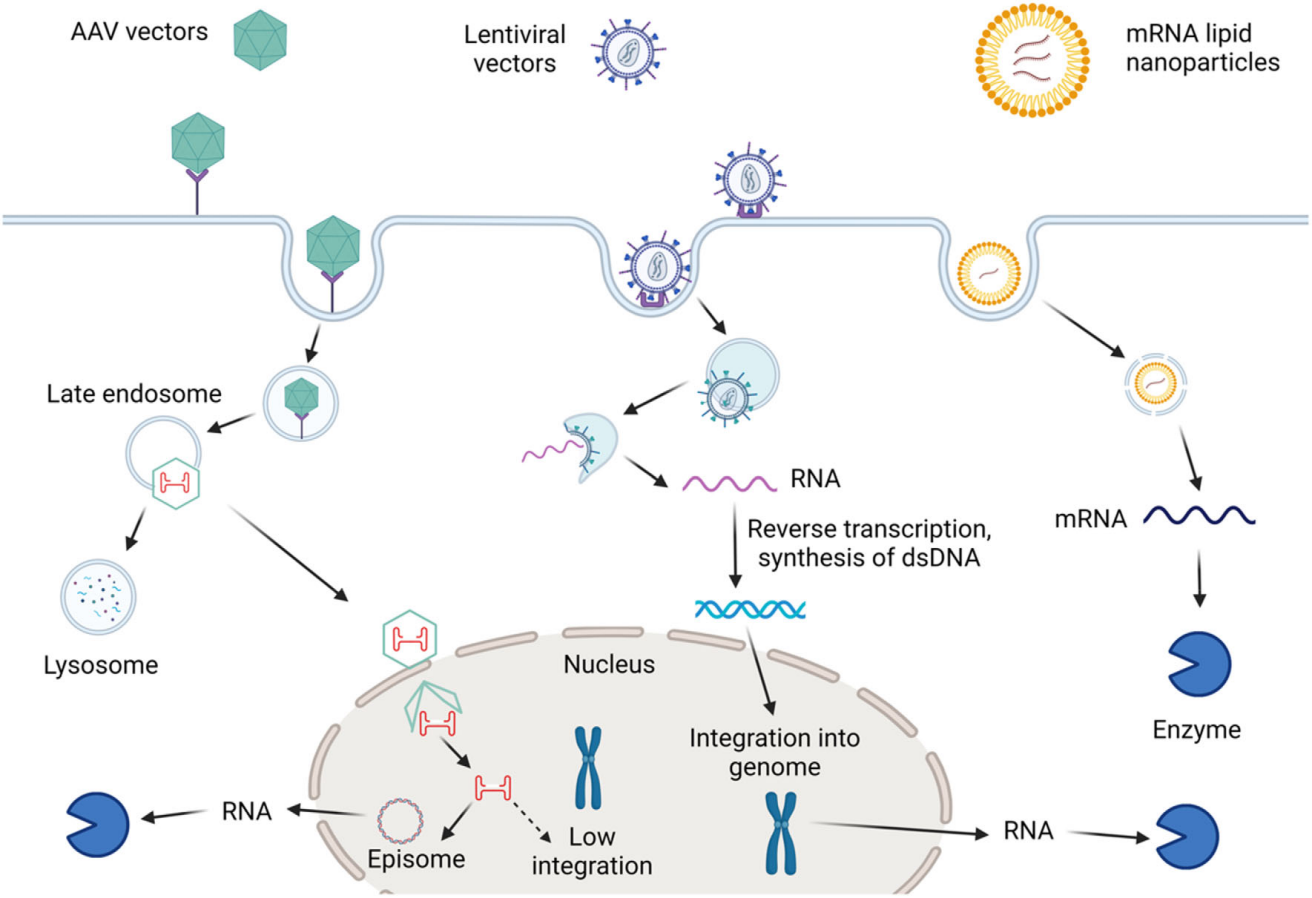
Transduction pathways of lentiviral, adeno‐associated viral vectors and messenger RNA (mRNA) lipid nanoparticles: cellular uptake and in‐cell processing. AAV, adeno‐associated virus.

AAV‐mediated gene transfer relies predominantly on episomes, circular double‐stranded DNA molecules present in the nucleus, which are diluted and lost during cell division^50^ (Figure 2). This was recognised as a major limitation in obtaining sustained transgene efficacy in the rapidly growing liver in young animals, unless considering AAV reinjection.^60^ While the correction of the adult phenotype of *Spf* ^*ash*^ mice was maintained overtime after systemic injection of a hepatotropic AAV8 vector encoding the murine *Otc* gene under the transcriptional control of a liver‐specific promoter, metabolic correction and the benefit of AAV gene transfer were rapidly lost in neonatal animals.^18^ Despite various preclinical attempts using different immunosuppressive protocols, successful AAV reinjection remains a challenge which is yet to be fully overcome.

Another question emerged regarding the potential dominant‐negative effect of mutant proteins on their wild‐type counterparts following gene transfer. After AAV8 transgene delivery of mutant *Otc* alleles to the wild‐type murine liver, mice exhibited no reduction in OTC activity regardless of the expression of the mutant proteins.^61^ This confirmed that the mutant proteins do not exert a dominant‐negative effect on wild‐type OTC, supporting translational efficacy of AAV gene transfer.

Within few years, the AAV gene addition strategy had become the leading and safest technology for liver targeting. Numerous preclinical successes in UCDs and other liver IMD had paved the way to early phase clinical trials over the next decade. However, the predominantly non‐integrative nature of AAV‐mediated gene transfer was rapidly recognised as a major hurdle for sustained efficacy in paediatric liver IMD, with re‐injection prevented by the anti‐AAV humoral immune response generated by the initial administration. Alternative strategies for paediatric UCDs had to be considered.

## 6 2010S AND 2020S: GENE EDITING, GENOMIC INTEGRATION AND NON‐VIRAL VECTORS

Over the past decade, gene addition has been the mainstay therapeutic strategy with AAV‐based vectors being the leading successfully translated liver‐targeting technology. Alternative strategies for transduction of the paediatric liver either via stable genomic transgene integration or via transient messenger RNA (mRNA) therapy, have shown proof of concept and are now being translated (Table 1).

**TABLE 1:** Clinical trials for ornithine transcarbamylase deficiency.

Sponsor	Phase	Status	Vector	NCT number (clinicaltrials.gov)
University of Pennsylvania	I	T	Adenoviral 5	NCT00004498
Translate Bio, Inc.	I/II	W	mRNA	NCT03767270
Arcturus Therapeutics, Inc.	Ia	C	mRNA	NCT04416126
Arcturus Therapeutics, Inc.	Ib	R	mRNA	NCT04442347
Arcturus Therapeutics, Inc.	III	R	mRNA	NCT05526066
Ultragenyx	I/II	C	AAV8	NCT02991144
Ultragenyx	III	R	AAV8	NCT05345171
University College London	I/II	NYR	AAV‐LK03	NCT05092685

Abbreviations: AAV, adeno‐associated virus; C, completed; NYR, not yet recruiting; messenger RNA, mRNA; R, recruiting; T, terminated; W, withdrawn.

## 7 NON‐INTEGRATIVE AAV GENE ADDITION

Following various proof of concept studies in preclinical models of UCDs, a first clinical trial CAPtivate of AAV‐mediated *OTC* gene addition using AAV8 and targeting late‐onset adult OTC deficient patients was sponsored by Ultragenyx (NCT02991144). This open‐label dose‐escalation pilot phase I/II clinical trial showed a marginal efficacy profile of DTX301 with a dose range of 3.2 × 10^12^–1.7 × 10^13^ GC/kg. Seven patients were considered responders out of 11 treated, with sustained effects up to 4 years for the longest‐treated responder.^62^ Four complete responder patients managed to discontinue their ammonia scavenger drugs and liberalised their diet. This programme has started a recruiting phase III clinical trial (NCT05345171).^63^ Another dose‐escalation open‐label dose‐escalation HORACE clinical trial targeting paediatric OTC‐deficient patients and sponsored by University College London (NCT05092685) is at the pre‐recruiting stage.^63^ This trial will use an engineered hepatotropic capsid AAV‐LK03 with a log‐higher efficacy in transducing human hepatocytes compared to AAV8.^64^ This better transduction rate of AAV‐LK03 and related AAV3B capsids was shown by different groups using a tyrosinaemic and immunodeficient *Fah* ^*−/−*^ */Rag2* ^*−/−*^ */Il2rgh* ^*−/−*^ (FRG) mouse model engrafted with primary human hepatocytes, thereby producing a chimeric mouse–human liver with repopulation of the mouse liver by >95% of human hepatocytes.^64^, ^65^, ^66^ Effectively recent haemophilia A phase I/II clinical trials suggested a better transduction with AAV‐LK03 versus AAV8 capsids with a relative higher expression of Factor VIII at similar doses, for example, 16%–194% (NCT03003533) versus 54%–69% (NCT03370172) Factor VIII activity at peak following injection of 6 × 10^12^ vg/kg in adults with severe haemophilia A, respectively.^67^, ^68^ HORACE preclinical studies have demonstrated safety in non‐human primates^69^ and a reduced seroprevalence for engineered versus wild‐type AAV capsids in humans.^70^

More recently, additional preclinical studies with AAV‐mediated gene addition have shown additional benefit in OTC‐deficient *Spf* ^*ash*^ mice, with long‐term benefit by preventing chronic liver disease and fibrosis.^71^ Multiple rounds of codon‐optimisation of the *OTC* transgene can significantly improve mRNA translatability, hence long‐term therapeutic efficacy with potential better safety if the injected AAV dose can be reduced for a similar effect.^72^ Following success in OTC deficiency, other UCDs such as CPS1 deficiency,^73^ ASS deficiency,^74^ ASL deficiency^12^, ^75^ and ARG1 deficiency^16^, ^76^, ^77^ have been targeted with AAV gene addition with success in adult mice, but limited efficacy in neonates. For CPS1 deficiency, the size of the transgene is too large to be packaged in the open reading frame of a single AAV vector. Therefore, the transgene was split and administered via two AAV vectors with an overlapping coding sequence enabling homologous recombination and synthesis of the active enzyme.^73^ Interestingly sequential injections of AAV8 gene therapy involving fetal, neonatal and additional post‐natal injections to treat an ASS‐deficient model with neonatal lethality required cross‐fostering of pups to vector‐naïve dams, as immunised dams with neutralising antibodies (NAbs) following fetal injections would pass these NAbs through milk, thereby blocking successful liver transduction.^74^ Gene therapy for ASL deficiency has also been investigated, using a codon‐optimised human *ASL* within AAV8 with targeted delivery to the liver in a hypomorphic mouse model. Administration via the temporal facial vein led to increased survival, but not to wild‐type levels. Intravenous injection in young adult mice led to correction of metabolites.^75^ Another study also utilised a single‐stranded AAV8 vector with murine *Asl* delivered systemically, leading to phenotypic rescue of the adult hypomorphic *Asl* ^*Neo/Neo*^ mouse model and limited correction of the neonatal animal after a single intravenous injection.^12^

## 8 INTEGRATIVE GENE ADDITION STRATEGIES

Due to the likely limitations of sustained efficacy of liver‐targeting AAV gene therapy in paediatrics, integrative strategies into the host genome have been developed to enable long‐term transgene expression.

### 8.1 Non‐targeted strategies

Random integration of the transgene of interest has been successfully achieved in UCDs with or without nuclease. A preclinical study using a *piggybac* transposase delivered by a dual AAV system providing the transgene and the nuclease was successfully tested in the ornithine transcarbamylase deficiency (OTCD) *Spf* ^*ash*^ and the neonatally lethal ASS‐deficient mouse models, enabling sustained phenotypic correction until adulthood following a single systemic neonatal injection.^78^

Another approach using in vivo lentiviral gene therapy has been successfully tested in ASLD providing long‐term cure of the ureagenesis defect following a single neonatal intravenous injection and without safety concerns.^79^ Lentiviral vectors are single‐stranded RNA vectors which release their cargo in the cytoplasm, where reverse transcription and synthesis of a second DNA strand enables nuclear migration and insertion into the genome (Figure 2). Integration carries a theoretical risk of insertional mutagenesis but has not been observed with lentiviral vectors in various preclinical models, for example, mouse, dog, and non‐human primate, following in vivo delivery.^80^, ^81^

### 8.2 Targeted strategies or genome editing

**FIGURE 3:**
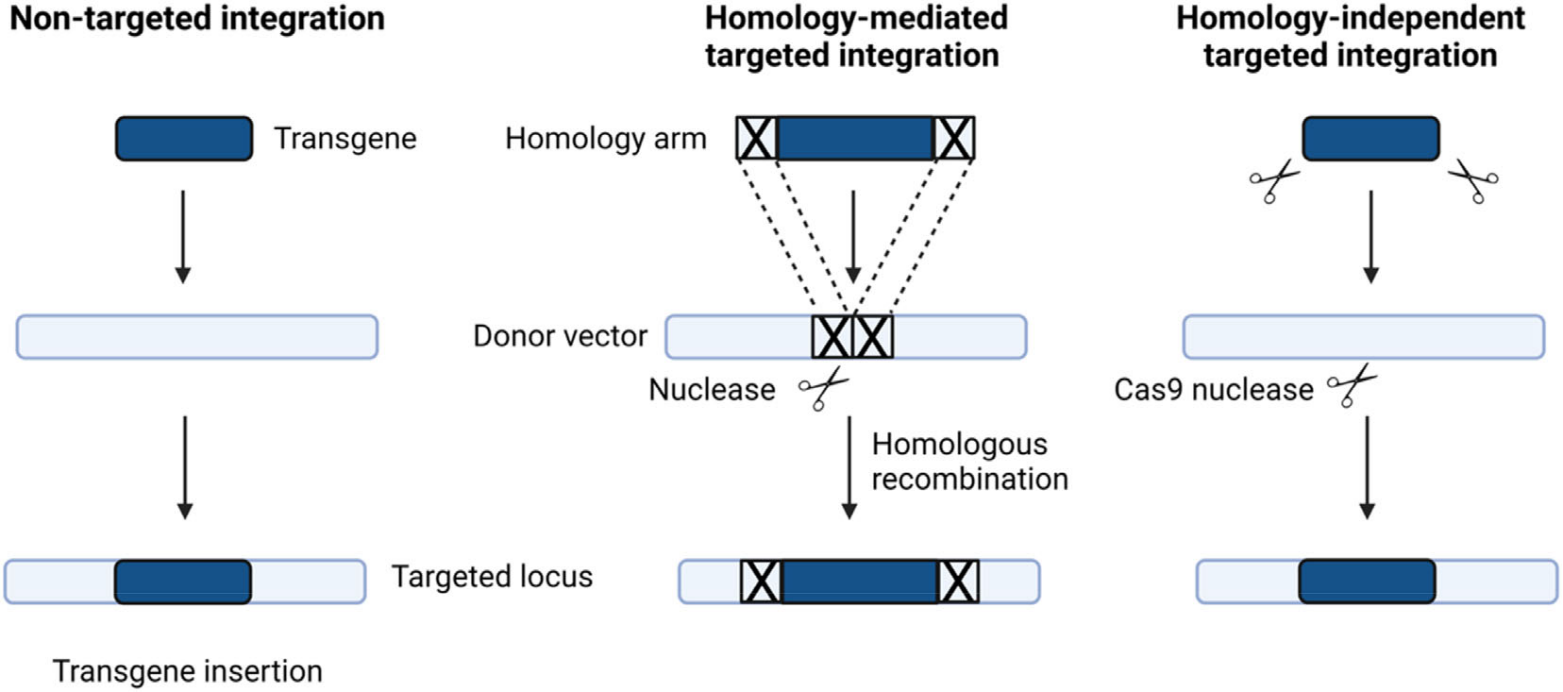
Non‐targeted and targeted integrations. Non‐targeted integration is mediated by integrative viral vector systems like lentiviral vectors or nucleases such as *piggybac* transposase. Targeted integration can use homologous recombination only but usually combines the action of a nuclease and homologous recombination, a strategy which shows a higher insertional rate. Homology‐independent targeted integration (HITI) does not use homology arms but a *Cas9* nuclease, which cuts the genomic target sequence and the donor plasmid DNA, thereby creating complementary blunt ends between both target and donor sequences. The donor DNA plasmid is used for repair by the non‐homologous end‐joining pathway and integrates at the genome double‐strand break site.

Targeted gene therapy can reduce the risk of tumorigenic events by choosing a genetic locus identified as a ‘safe harbour’. This is achieved with or without nuclease, often by homologous recombination strategy (Figure 3).

UCDs share a large diversity of genotypes with numerous private mutations and few hotspots.^82^, ^83^, ^84^ Although gene editing of a single missense mutation has been successfully achieved in vivo, this is not a reliable translational therapy for these disorders. Therefore, the vast efforts are focussing on whole transgene integration.

Promoterless and nuclease‐free gene replacement by homologous recombination has shown proof of concept in numerous liver IMD, for example, haemophilia B,^85^ Crigler–Najjar,^86^ methylmalonic acidaemia^87^ and is being assessed for safety and efficacy in a phase I/II clinical trial SUNRISE (NCT04581785) for methylmalonic acidaemia sponsored by LogicBio Therapeutics.^63^ Depending on the target site in the genome, the absence of enhancer/promoter might enable physiological regulation of the gene by an endogenous promoter and reduce the risk of transactivation, which has been identified as one of the main risks of insertional mutagenesis.^59^

Nuclease‐mediated genome editing relies on the use of a nuclease, for example, TALENS, Zinc Fingers Nucleases, meganuclease and *Cas9* editing the host genome at a specific locus, which is achieved by a single guide RNA (sgRNA) specifically recognising a 20 bp‐long DNA sequence. The nuclease creates a DNA single or double‐strand break, which is then repaired by homology‐directed repair (HDR) based on a donor DNA template. A dual AAV system delivers the sgRNA with the donor *OTC* template and the *Cas9*. This approach has been used in the OTCD *Spf* ^*ash*^ mouse to correct successfully the missense mutation of this mouse model, with a better efficacy in neonatal animals compared to young adults, with 15% versus 6% of hepatocytes transduced at 3 weeks post‐injection, respectively.^88^

Another approach combining DNA double‐strand break and homologous recombination via two 900 bp‐long homologous arms flanking the *OTC* donor template was then successfully tested using the same sgRNA via a similar dual AAV approach injected systemically in neonatal OTCD *Spf* ^*ash*^ pups. Twenty‐five per cent of hepatocytes were successfully transduced at 3 weeks post‐injection with correction of the phenotype. However, this was associated with a 28% rate of insertions and deletions due to limited HDR and a repair of the DNA double‐strand break by non‐homologous end joining (NHEJ).^89^ In a similar approach, CRISPR‐*Cas9* cleavage combined with HDR was used in vivo to correct missense mutations of engrafted patient‐derived OTCD primary hepatocytes in FRG mice. The delivery of the CRISPR‐*Cas9* technology was achieved by a dual AAV system with an engineered hepatotropic AAV‐NP59 capsid. This approach led to an unparalleled level of correction, with up to 29% of hepatocytes corrected.^90^ This same approach of *Cas9*‐mediated gene insertion coupled with homologous recombination was tested in ARG1‐deficient induced pluripotent stem cells (iPS)‐derived hepatocytes with insertion of the full‐length codon‐optimised human ARG1 cDNA at the exon 1 of the hypoxanthine‐guanine phosphoribosyltransferase locus. This successfully restored arginase activity, and thus ureagenesis.^91^ This approach has been tried in CPS1 deficiency with targeted integration via *Cas9* cut and homologous recombination‐mediated gene insertion at the AAVS1 locus. However, edited cells failed to show restoration of ureagenesis in vitro.^92^ The rapid development of iPSC‐derived hepatocytes and liver organoids is facilitating the screening of editing strategies in vitro.^93^

Whole transgene insertion driven by combined DNA double‐strand break and homologous recombination was recently described with an alternative nuclease, the ARCUS meganuclease. ARCUS is based on a naturally occurring homodimeric genome editing enzyme I‐CreI, which comes from the *Chlamydomonas reinhardtii* chloroplast genome and allows high‐precision double‐stranded DNA cuts.^94^ The ARCUS meganuclease showed a high rate of edited hepatocytes (16%) at the exon 7 of the *PCSK9* gene identified as a safe harbour. Sustained efficacy after a single systemic injection in OTCD *Spf* ^*ash*^ pups was observed over a year post‐injection. The safety of this editing strategy is being validated in non‐human primates with satisfactory preliminary results.^95^

Although these preclinical successes are encouraging, there are a number of translational challenges that are yet to be fully addressed in terms of both efficacy and safety. For example, it is not yet clear that HDR‐dependent approaches will be sufficiently efficient in the human liver in vivo, even in early infancy where the liver is growing rapidly, and approaches exploiting NHEJ, which is cell cycle independent, may be required.^96^ Regarding safety, a major incompletely resolved consideration is the frequency and nature of unintended on‐ and off‐target editing events with genotoxic potential.

Another approach of targeted integration is called homology‐independent targeted integration, which requires a *Cas9* nuclease but without homology arms. The *Cas9* nuclease cut *Cas9* target sites both in the genomic target sequence and a circular plasmid FNA template. This creates blunt extremities both at the genomic target site and the extremities of the linearized donor sequence, which enables an integration of the donor DNA plasmid at the genome double‐strand break site using a NHEJ pathway for repair (Figure 3).^96^ This approach has not been tried with UCDs.

## 9 mRNA THERAPY

The safety and efficacy of mRNA technology have been demonstrated worldwide during the COVID‐19 pandemic with a new vaccine platform against SarS‐CoV‐2 based on mRNA administered to millions of individuals with higher efficacy compared to viral vector or recombinant protein vaccine platforms.^97^ Greater than 400 RNA‐related products are in development with an exponential increase in investment.^98^ Different RNA technologies have been tested with mRNA addition or gene silencing using regulatory RNAs, that is, small interfering or short hairpin RNA. Lipid nanoparticles (LNPs) containing RNA are taken up and conveyed via endosomes for release of their cargo in the cytoplasm (Figure 2). Due to their transient effect, re‐administration is necessary for long‐term efficacy. Messenger RNA is encapsulated in LNPs to prevent rapid degradation by RNases. Neither appropriately modified mRNA nor LNP has been reported to trigger sustained immune responses,^99^ enabling successful re‐administration. Greater than 30 RNA‐based products are now approved or in late‐phase clinical trials for vaccines, cancer immunotherapies, gene editing or protein replacement therapies for inherited liver or neuromuscular disorders.^100^, ^101^

This approach has been successfully explored for ARG1 deficiency in a conditional knockout model of *ARG1* deficiency.^102^ Repeated dosing of *ARG1* mRNA led to sustained correction of the disease phenotype with normalisation of plasma ammonia and arginine levels without toxicity. This approach enabled the description and the correction of specific neurological pathophysiological features of the disease, that is, dysmyelination of the central nervous system, particularly the corticospinal tracts, sparing the peripheral nervous system.^31^ Messenger RNA therapy was also successfully assessed in a mouse model of *ASL* deficiency, treating neonates and rescuing adult animals with correction of biomarkers to physiological levels.^103^ This showed a correction of the chronic liver disease observed in ASLD and restoration of physiological glutathione metabolism.

Messenger RNA therapy for OTCD (ARCT‐810) has been tested in phase Ia (NCT04416126) and Ib (NCT04442347) clinical trials with a satisfactory safety profile.^104^ This programme sponsored by Arcturus Therapeutics is currently in a phase II clinical trial (NCT05526066) recruiting adolescent and adult OTC‐deficient patients.^63^

## 10 CONCLUSION

Gene therapy for UCDs is rapidly expanding. Due to the severity of the clinical phenotype, favourable risk–benefit profile for experimental intervention and relatively common presentation in the field of IMD, most pioneering liver‐targeting technologies will explore applications as proof of concept in UCDs. Initial signs of translational promise in early‐phase clinical trials in late‐onset OTC‐deficient adult patients are paving the way for a wider development of gene therapy for younger patients and for all UCDs. For safety and efficacy reasons, novel integrative and transient technologies are actively being developed to achieve the holy grail, complete and stable phenotypic correction from the neonatal period in the sickest early‐onset patients.

## FUNDING INFORMATION

This work was supported by funding from the United Kingdom Medical Research Council Clinician Scientist Fellowship MR/T008024/1 and NIHR Great Ormond Street Hospital Biomedical Research Centre to JB and Project Grant funding (423400) from the Australian National Health and Medical Research Council to IA. The views expressed are those of the author(s) and not necessarily those of the NHS, the NIHR or the Department of Health.

## CONFLICT OF INTEREST STATEMENT

Julien Baruteau is a consultant for biopharmaceutical companies developing therapies for urea cycle defects and has a collaborative research agreement with Moderna Therapeutics. Claire Duff and Ian E. Alexander have no conflicts of interest to declare.

## ETHICS STATEMENT

This review article does not require ethics approval.

## Data Availability

In this review, there are no data to share.

## References

[1] Gropman AL , Batshaw ML . Cognitive outcome in urea cycle disorders. Mol Genet Metab. 2004;81(Suppl 1):S58‐S62. doi:10.1016/j.ymgme.2003.11.016 15050975

[2] Summar ML , Koelker S , Freedenberg D , et al. The incidence of urea cycle disorders. Mol Genet Metab. 2013;110:179‐180. doi:10.1016/j.ymgme.2013.07.008 23972786 PMC4364413

[3] Nettesheim S , Kölker S , Karall D , et al. Incidence, disease onset and short‐term outcome in urea cycle disorders–cross‐border surveillance in Germany, Austria and Switzerland. Orphanet J Rare Dis. 2017;12:111. doi:10.1186/s13023-017-0661-x 28619060 PMC5472961

[4] Ah Mew N , Simpson KL , Gropman AL , et al. Urea cycle disorders overview. In: Adam MP , Mirzaa GM , Pagon RA , eds. et al.GeneReviews([R]). University of Washington, Seattle; 2017. 20301396

[5] Nassogne MC , Heron B , Touati G , Rabier D , Saudubray JM . Urea cycle defects: management and outcome. J Inherit Metab Dis. 2005;28:407‐414. doi:10.1007/s10545-005-0303-7 15868473

[6] Gebhardt R , Matz‐Soja M . Liver zonation: novel aspects of its regulation and its impact on homeostasis. World J Gastroenterol. 2014;20:8491‐8504. doi:10.3748/wjg.v20.i26.8491 25024605 PMC4093700

[7] Baruteau J , Diez‐Fernandez C , Lerner S , et al. Argininosuccinic aciduria: recent pathophysiological insights and therapeutic prospects. J Inherit Metab Dis. 2019;42:1147‐1161. doi:10.1002/jimd.12047 30723942

[8] Stettner N , Rosen C , Bernshtein B , et al. Induction of nitric‐oxide metabolism in enterocytes alleviates colitis and inflammation‐associated colon cancer. Cell Rep. 2018;23:1962‐1976. doi:10.1016/j.celrep.2018.04.053 29768197 PMC5976577

[9] Premkumar MH , Sule G , Nagamani SC , et al. Argininosuccinate lyase in enterocytes protects from development of necrotizing enterocolitis. Am J Physiol Gastrointest Liver Physiol. 2014;307:G347‐G354. doi:10.1152/ajpgi.00403.2013 24904080 PMC4121640

[10] Kho J , Tian X , Wong WT , et al. Argininosuccinate lyase deficiency causes an endothelial‐dependent form of hypertension. Am J Hum Genet. 2018;103:276‐287. doi:10.1016/j.ajhg.2018.07.008 30075114 PMC6080833

[11] Jin Z , Kho J , Dawson B , et al. Nitric oxide modulates bone anabolism through regulation of osteoblast glycolysis and differentiation. J Clin Invest. 2021;131. doi:10.1172/JCI138935 PMC7919726 33373331

[12] Baruteau J , Perocheau DP , Hanley J , et al. Argininosuccinic aciduria fosters neuronal nitrosative stress reversed by Asl gene transfer. Nat Commun. 2018;9:3505. doi:10.1038/s41467-018-05972-1 30158522 PMC6115417

[13] Lerner S , Eilam R , Adler L , et al. ASL expression in ALDH1A1(+) neurons in the substantia nigra metabolically contributes to neurodegenerative phenotype. Hum Genet. 2021;140:1471‐1485. doi:10.1007/s00439-021-02345-5 34417872 PMC8460544

[14] Diez‐Fernandez C , Hertig D , Loup M , et al. Argininosuccinate neurotoxicity and prevention by creatine in argininosuccinate lyase deficiency: an in vitro study in rat three‐dimensional organotypic brain cell cultures. J Inherit Metab Dis. 2019;42:1077‐1087. doi:10.1002/jimd.12090 30907007

[15] Haberle J , Burlina A , Chakrapani A , et al. Suggested guidelines for the diagnosis and management of urea cycle disorders: first revision. J Inherit Metab Dis. 2019;42:1192‐1230. doi:10.1002/jimd.12100 30982989

[16] Hu C , Tai DS , Park H , et al. Minimal ureagenesis is necessary for survival in the murine model of hyperargininemia treated by AAV‐based gene therapy. Gene Ther. 2015;22:111‐115. doi:10.1038/gt.2014.106 25474440 PMC4320015

[17] Baruteau JP , Perocheau DP , Hanley J , et al. Argininosuccinic aciduria fosters neuronal nitrosative stress reversed by Asl gene transfer. Nat Commun, In Press. 2018;9:3505. 10.1038/s41467-018-05972-1 PMC6115417 30158522

[18] Cunningham SC , Spinoulas A , Carpenter KH , Wilcken B , Kuchel PW , Alexander IE . AAV2/8‐mediated correction of OTC deficiency is robust in adult but not neonatal Spf(ash) mice. Mol Ther. 2009;17:1340‐1346. doi:10.1038/mt.2009.88 19384294 PMC2835243

[19] Ban K , Sugiyama N , Sugiyama K , et al. A pediatric patient with classical citrullinemia who underwent living‐related partial liver transplantation. Transplantation. 2001;71:1495‐1497. 11391244 10.1097/00007890-200105270-00026

[20] Bachmann C . Mechanisms of hyperammonemia. Clin Chem Lab Med. 2002;40:653‐662. doi:10.1515/CCLM.2002.112 12241009

[21] Nathwani AC , Reiss UM , Tuddenham EGD , et al. Long‐term safety and efficacy of factor IX gene therapy in hemophilia B. N Engl J Med. 2014;371:1994‐2004. doi:10.1056/NEJMoa1407309 25409372 PMC4278802

[22] Squires RH , Ng V , Romero R , et al. Evaluation of the pediatric patient for liver transplantation: 2014 practice guideline by the American Association for the Study of Liver Diseases, American Society of Transplantation and the North American Society for Pediatric Gastroenterology, Hepatology, and Nutrition. J Pediatr Gastroenterol Nutr. 2014;59:112‐131. doi:10.1097/MPG.0000000000000431 25222807

[23] Ziogas IA , Wu WK , Matsuoka LK , et al. Liver transplantation in children with urea cycle disorders: the importance of minimizing waiting time. Liver Transplant. 2021;27:1799‐1810. doi:10.1002/lt.26186 PMC9291867 34058057

[24] Yu L , Rayhill SC , Hsu EK , Landis CS . Liver transplantation for urea cycle disorders: analysis of the united network for organ sharing database. Transplant Proc. 2015;47:2413‐2418. doi:10.1016/j.transproceed.2015.09.020 26518943

[25] Mori T , Nagai K , Mori M , et al. Progressive liver fibrosis in late‐onset argininosuccinate lyase deficiency. Pediatr Dev Pathol. 2002;5:597‐601. doi:10.1007/s10024-002-0109-7 12370774

[26] Zimmermann A , Bachmann C , Baumgartner R . Severe liver fibrosis in argininosuccinic aciduria. Arch Pathol Lab Med. 1986;110:136‐140. 3753845

[27] Yaplito‐Lee J , Chow CW , Boneh A . Histopathological findings in livers of patients with urea cycle disorders. Mol Genet Metab. 2013;108:161‐165. doi:10.1016/j.ymgme.2013.01.006 23403242

[28] Marble M , McGoey RR , Mannick E , et al. Living related liver transplant in a patient with argininosuccinic aciduria and cirrhosis: metabolic follow‐up. J Pediatr Gastroenterol Nutr. 2008;46:453‐456. doi:10.1097/MPG.0b013e3180ca8720 18367960

[29] Gucer S , Asan E , Atilla P , Tokatli A , Caglar M . Early cirrhosis in a patient with type I citrullinaemia (CTLN1). J Inherit Metab Dis. 2004;27:541‐542. doi:10.1023/b:boli.0000037401.63596.de 15334737

[30] Baruteau J , Jameson E , Morris AA , et al. Expanding the phenotype in argininosuccinic aciduria: need for new therapies. J Inherit Metab Dis. 2017;40:357‐368. doi:10.1007/s10545-017-0022-x 28251416 PMC5393288

[31] Liu XB , Haney JR , Cantero G , et al. Hepatic arginase deficiency fosters dysmyelination during postnatal CNS development. JCI Insight. 2019;4:e130260. doi:10.1172/jci.insight.130260 PMC6777909 31484827

[32] Ricciuti FC , Gelehrter TD , Rosenberg LE . X‐chromosome inactivation in human liver: confirmation of X‐linkage of ornithine transcarbamylase. Am J Hum Genet. 1976;28:332‐338. 941900 PMC1685051

[33] McCullough BA , Yudkoff M , Batshaw ML , Wilson JM , Raper SE , Tuchman M . Genotype spectrum of ornithine transcarbamylase deficiency: correlation with the clinical and biochemical phenotype. Am J Med Genet. 2000;93:313‐319. doi:10.1002/1096-8628(20000814)93:4<313::aid-ajmg11>3.0.co;2-m 10946359

[34] Kok CY , Cunningham SC , Kuchel PW , Alexander IE . Insights into gene therapy for urea cycle defects by mathematical modeling. Hum Gene Ther. 2019;30:1385‐1394. doi:10.1089/hum.2019.053 31215258

[35] Jungermann K . Zonation of metabolism and gene expression in liver. Histochem Cell Biol. 1995;103:81‐91. 7634156 10.1007/BF01454004

[36] Cabanes‐Creus M , Navarro RG , Liao SHY , et al. Characterization of the Humanized FRG Mouse Model and Development of an AAVLK03 Variant with Improved Liver Lobular Biodistribution. Mol Ther Methods Clin Dev. 2023;28:220‐237. 10.1016/j.omtm.2022.12.014 PMC9860073 36700121

[37] Lautt WW , Greenway CV . Conceptual review of the hepatic vascular bed. Hepatology. 1987;7:952‐963. doi:10.1002/hep.1840070527 3308669

[38] Braet F , Wisse E . Structural and functional aspects of liver sinusoidal endothelial cell fenestrae: a review. Comp Hepatol. 2002;1:1. doi:10.1186/1476-5926-1-1 12437787 PMC131011

[39] Neumann UP , Guckelberger O , Langrehr JM , et al. Impact of human leukocyte antigen matching in liver transplantation. Transplantation. 2003;75:132‐137. doi:10.1097/00007890-200301150-00024 12544885

[40] Mingozzi F , High KA . Immune responses to AAV in clinical trials. Curr Gene Ther. 2011;11:321‐330. 21557723 10.2174/156652311796150354

[41] Piccolo P , Brunetti‐Pierri N . Challenges and prospects for helper‐dependent adenoviral vector‐mediated gene therapy. Biomedicine. 2014;2:132‐148. 10.3390/biomedicines2020132 PMC5423471 28548064

[42] Kotterman MA , Chalberg TW , Schaffer DV . Viral vectors for gene therapy: translational and clinical outlook. Annu Rev Biomed Eng. 2015;17:63‐89. doi:10.1146/annurev-bioeng-071813-104938 26643018

[43] Nagamani SC , Campeau PM , Shchelochkov OA , et al. Nitric‐oxide supplementation for treatment of long‐term complications in argininosuccinic aciduria. Am J Hum Genet. 2012;90:836‐846. doi:10.1016/j.ajhg.2012.03.018 22541557 PMC3376491

[44] Mian A , Lee B . Urea‐cycle disorders as a paradigm for inborn errors of hepatocyte metabolism. Trends Mol Med. 2002;8:583‐589. 12470992 10.1016/s1471-4914(02)02437-1

[45] Ye X , Whiteman B , Jerebtsova M , Batshaw ML . Correction of argininosuccinate synthetase (AS) deficiency in a murine model of citrullinemia with recombinant adenovirus carrying human AS cDNA. Gene Ther. 2000;7:1777‐1782. doi:10.1038/sj.gt.3301303 11083500

[46] Mian A , McCormack WM Jr , Mane V , et al. Long‐term correction of ornithine transcarbamylase deficiency by WPRE‐mediated overexpression using a helper‐dependent adenovirus. Mol Ther. 2004;10:492‐499. doi:10.1016/j.ymthe.2004.05.036 15336649

[47] Gau CL , Rosenblatt RA , Cerullo V , et al. Short‐term correction of arginase deficiency in a neonatal murine model with a helper‐dependent adenoviral vector. Mol Ther. 2009;17:1155‐1163. doi:10.1038/mt.2009.65 19367256 PMC2835205

[48] Raper SE , Chirmule N , Lee FS , et al. Fatal systemic inflammatory response syndrome in a ornithine transcarbamylase deficient patient following adenoviral gene transfer. Mol Genet Metab. 2003;80:148‐158. 14567964 10.1016/j.ymgme.2003.08.016

[49] Wilson JM . Lessons learned from the gene therapy trial for ornithine transcarbamylase deficiency. Mol Genet Metab. 2009;96:151‐157. doi:10.1016/j.ymgme.2008.12.016 19211285

[50] Baruteau J , Waddington SN , Alexander IE , Gissen P . Gene therapy for monogenic liver diseases: clinical successes, current challenges and future prospects. J Inherit Metab Dis. 2017;40:497‐517. doi:10.1007/s10545-017-0053-3 28567541 PMC5500673

[51] Nault JC , Datta S , Imbeaud S , et al. Recurrent AAV2‐related insertional mutagenesis in human hepatocellular carcinomas. Nat Genet. 2015;47:1187‐1193. doi:10.1038/ng.3389 26301494

[52] Logan GJ , Dane AP , Hallwirth CV , et al. Identification of liver‐specific enhancer‐promoter activity in the 3′ untranslated region of the wild‐type AAV2 genome. Nat Genet. 2017;49:1267‐1273. doi:10.1038/ng.3893 28628105

[53] Zhang J , Yu X , Herzog RW , Samulski RJ , Xiao W . Flies in the ointment: AAV vector preparations and tumor risk. Mol Ther. 2021;29:2637‐2639. doi:10.1016/j.ymthe.2021.08.016 34450107 PMC8417912

[54] de Jong YP , Herzog RW . AAV and hepatitis: cause or coincidence? Mol Ther. 2022;30:2875‐2876. doi:10.1016/j.ymthe.2022.08.001 35981546 PMC9482007

[55] Moscioni D , Morizono H , McCarter RJ , et al. Long‐term correction of ammonia metabolism and prolonged survival in ornithine transcarbamylase‐deficient mice following liver‐directed treatment with adeno‐associated viral vectors. Mol Ther. 2006;14:25‐33. doi:10.1016/j.ymthe.2006.03.009 16677864

[56] Bell P , Moscioni AD , McCarter RJ , et al. Analysis of tumors arising in male B6C3F1 mice with and without AAV vector delivery to liver. Mol Ther. 2006;14:34‐44. doi:10.1016/j.ymthe.2006.03.008 16682254

[57] Koeberl DD . Vector‐related tumorigenesis not found in ornithine transcarbamylase‐deficient mice. Mol Ther. 2006;14:1‐2. doi:10.1016/j.ymthe.2006.05.011 16750655

[58] Donsante A , Miller DG , Li Y , et al. AAV vector integration sites in mouse hepatocellular carcinoma. Science. 2007;317:477. doi:10.1126/science.1142658 17656716

[59] Chandler RJ , LaFave MC , Varshney GK , et al. Vector design influences hepatic genotoxicity after adeno‐associated virus gene therapy. J Clin Invest. 2015;125:870‐880. doi:10.1172/JCI79213 25607839 PMC4319425

[60] Cunningham SC , Dane AP , Spinoulas A , Alexander IE . Gene delivery to the juvenile mouse liver using AAV2/8 vectors. Mol Ther. 2008;16:1081‐1088. doi:10.1038/mt.2008.72 28178471

[61] Ginn SL , Cunningham SC , Zheng M , Spinoulas A , Carpenter KH , Alexander IE . In vivo assessment of mutations in OTC for dominant‐negative effects following rAAV2/8‐mediated gene delivery to the mouse liver. Gene Ther. 2009;16:820‐823. doi:10.1038/gt.2009.38 19357713

[62] Harding CO , Geberhiwot T , Couce ML , et al. Safety and efficacy of DTX301 in adults with late‐onset ornithine transcarbamylase (OTC) deficiency: a phase 1/2 trial. Mol Ther. 2022;30:219.

[63] Clinicaltrials.gov.

[64] Lisowski L , Dane AP , Chu K , et al. Selection and evaluation of clinically relevant AAV variants in a xenograft liver model. Nature. 2014;506:382‐386. doi:10.1038/nature12875 24390344 PMC3939040

[65] Vercauteren K , Hoffman BE , Zolotukhin I , et al. Superior In vivo transduction of human hepatocytes using engineered AAV3 capsid. Mol Ther. 2016;24:1042‐1049. doi:10.1038/mt.2016.61 27019999 PMC4923326

[66] Wang L , Bell P , Somanathan S , et al. Comparative study of liver gene transfer with AAV vectors based on natural and engineered AAV capsids. Mol Ther. 2015;23:1877‐1887. doi:10.1038/mt.2015.179 26412589 PMC4700115

[67] George LA , Monahan PE , Eyster ME , et al. Multiyear factor VIII expression after AAV gene transfer for hemophilia A. N Engl J Med. 2021;385:1961‐1973. doi:10.1056/NEJMoa2104205 34788507 PMC8672712

[68] Chapin JA , Allen G , Álvarez‐Román MT , et al. Results from a phase 1/2 safety and dose escalation study of TAK‐754, an AAV8 vector with a codon‐optimized B‐domain‐deleted factor VIII transgene in severe Hemophilia A. Hematology. 2021;27:122.

[69] Baruteau J , Cunningham SC , Yilmaz BS , et al. Safety and efficacy of an engineered hepatotropic AAV gene therapy for ornithine transcarbamylase deficiency in cynomolgus monkeys. Mol Ther Methods Clin Dev. 2021;23:135‐146. doi:10.1016/j.omtm.2021.09.005 34703837 PMC8517016

[70] Perocheau D , Cunningham S , Lee J , et al. Age‐related seroprevalence of antibodies against AAV‐LK03 in a UK population cohort. Hum Gene Ther. 2018;30:79‐87. doi:10.1089/hum.2018.098 30027761 PMC6343184

[71] Wang L , Bell P , Morizono H , et al. AAV gene therapy corrects OTC deficiency and prevents liver fibrosis in aged OTC‐knock out heterozygous mice. Mol Genet Metab. 2017;120:299‐305. doi:10.1016/j.ymgme.2017.02.011 28283349 PMC5423267

[72] De Sabbata G , Boisgerault F , Guarnaccia C , et al. Long‐term correction of ornithine transcarbamylase deficiency in Spf‐ash mice with a translationally optimized AAV vector. Mol Ther Methods Clin Dev. 2021;20:169‐180. doi:10.1016/j.omtm.2020.11.005 33473356 PMC7786024

[73] Nitzahn M , Allegri G , Khoja S , et al. Split AAV‐mediated gene therapy restores ureagenesis in a murine model of carbamoyl phosphate synthetase 1 deficiency. Mol Ther. 2020;28:1717‐1730. doi:10.1016/j.ymthe.2020.04.011 32359471 PMC7335736

[74] Kok CY , Cunningham SC , Carpenter KH , et al. Adeno‐associated virus‐mediated rescue of neonatal lethality in argininosuccinate synthetase‐deficient mice. Mol Ther. 2013;21:1823‐1831. doi:10.1038/mt.2013.139 23817206 PMC3808136

[75] Ashley SN , Nordin JML , Buza EL , Greig JA , Wilson JM . Adeno‐associated viral gene therapy corrects a mouse model of argininosuccinic aciduria. Mol Genet Metab. 2018;125:241‐250. doi:10.1016/j.ymgme.2018.08.013 30253962

[76] Lee EK , Hu C , Bhargava R , et al. Long‐term survival of the juvenile lethal arginase‐deficient mouse with AAV gene therapy. Mol Ther. 2012;20:1844‐1851. doi:10.1038/mt.2012.129 22760543 PMC3464644

[77] Lee EK , Hu C , Bhargava R , et al. AAV‐based gene therapy prevents neuropathology and results in normal cognitive development in the hyperargininemic mouse. Gene Ther. 2013;20:785‐796. doi:10.1038/gt.2012.99 23388701 PMC3679314

[78] Cunningham SC , Siew SM , Hallwirth CV , et al. Modeling correction of severe urea cycle defects in the growing murine liver using a hybrid recombinant adeno‐associated virus/piggyBac transposase gene delivery system. Hepatology. 2015;62:417‐428. doi:10.1002/hep.27842 26011400

[79] Touramanidou L , Gurung S , Cozmescu AC , et al. In vivo lentiviral gene therapy for argininosuccinic aciduria. Eur Soc Gene Cell Ther (ESGCT) Virtual Congr. 2021;32:A1‐A152.

[80] Cantore A , Ranzani M , Bartholomae CC , et al. Liver‐directed lentiviral gene therapy in a dog model of hemophilia B. Sci Transl Med. 2015;7:277ra228. doi:10.1126/scitranslmed.aaa1405 PMC5669486 25739762

[81] Milani M , Canepari C , Liu T , et al. Liver‐directed lentiviral gene therapy corrects hemophilia A mice and achieves normal‐range factor VIII activity in non‐human primates. Nat Commun. 2022;13:2454. doi:10.1038/s41467-022-30102-3 35508619 PMC9068791

[82] Caldovic L , Abdikarim I , Narain S , Tuchman M , Morizono H . Genotype‐phenotype correlations in ornithine transcarbamylase deficiency: a mutation update. J Genet Genom Yi Chuan Xue Bao. 2015;42:181‐194. doi:10.1016/j.jgg.2015.04.003 PMC4565140 26059767

[83] Balmer C , Pandey AV , Rüfenacht V , et al. Mutations and polymorphisms in the human argininosuccinate lyase (ASL) gene. Hum Mutat. 2014;35:27‐35. doi:10.1002/humu.22469 24166829

[84] Diez‐Fernandez C , Rufenacht V , Haberle J . Mutations in the human Argininosuccinate synthetase (ASS1) gene, impact on patients, common changes, and structural considerations. Hum Mutat. 2017;38:471‐484. doi:10.1002/humu.23184 28111830

[85] Barzel A , Paulk NK , Shi Y , et al. Promoterless gene targeting without nucleases ameliorates haemophilia B in mice. Nature. 2015;517:360‐364. doi:10.1038/nature13864 25363772 PMC4297598

[86] Porro F , Bortolussi G , Barzel A , et al. Promoterless gene targeting without nucleases rescues lethality of a Crigler‐Najjar syndrome mouse model. EMBO mol Med. 2017;9:1346‐1355. doi:10.15252/emmm.201707601 28751579 PMC5623861

[87] Chandler RJ , Venturoni LE , Liao J , et al. Promoterless, nuclease‐free genome editing confers a growth advantage for corrected hepatocytes in mice with methylmalonic acidemia. Hepatology. 2021;73:2223‐2237. doi:10.1002/hep.31570 32976669 PMC8252383

[88] Yang Y , Wang L , Bell P , et al. A dual AAV system enables the Cas9‐mediated correction of a metabolic liver disease in newborn mice. Nat Biotechnol. 2016;34:334‐338. doi:10.1038/nbt.3469 26829317 PMC4786489

[89] Wang L , Yang Y , Breton C , et al. A mutation‐independent CRISPR‐Cas9‐mediated gene targeting approach to treat a murine model of ornithine transcarbamylase deficiency. Sci Adv. 2020;6:eaax5701. doi:10.1126/sciadv.aax5701 32095520 PMC7015695

[90] Ginn SL , Amaya AK , Liao SHY , et al. Efficient in vivo editing of OTC‐deficient patient‐derived primary human hepatocytes. JHEP Rep. 2020;2:100065. doi:10.1016/j.jhepr.2019.100065 32039406 PMC7005564

[91] Lee PC , Truong B , Vega‐Crespo A , et al. Restoring ureagenesis in hepatocytes by CRISPR/Cas9‐mediated genomic addition to arginase‐deficient induced pluripotent stem cells. Mol Ther Nucleic Acids. 2016;5:e394. doi:10.1038/mtna.2016.98 PMC5155330 27898091

[92] Nitzahn M , Truong B , Khoja S , et al. CRISPR‐mediated genomic addition to CPS1 deficient iPSCs is insufficient to restore nitrogen homeostasis. Yale J Biol Med. 2021;94:545‐557. 34970092 PMC8686786

[93] Duff C , Baruteau J . Modelling urea cycle disorders using iPSCs. NPJ Regen Med. 2022;7:56. doi:10.1038/s41536-022-00252-5 36163209 PMC9513077

[94] Zekonyte U , Bacman SR , Smith J , et al. Mitochondrial targeted meganuclease as a platform to eliminate mutant mtDNA in vivo. Nat Commun. 2021;12:3210. doi:10.1038/s41467-021-23561-7 34050192 PMC8163834

[95] Wang L , Warzecha CC , Ralph KM , et al. AAV‐meganuclease‐mediated gene targeting achieves efficient and sustained transduction in newborn and infant macaque liver. Mol Ther. 2022;30:381.

[96] Suzuki K , Tsunekawa Y , Hernandez‐Benitez R , et al. In vivo genome editing via CRISPR/Cas9 mediated homology‐independent targeted integration. Nature. 2016;540:144‐149. doi:10.1038/nature20565 27851729 PMC5331785

[97] Zhang Z , Mateus J , Coelho CH , et al. Humoral and cellular immune memory to four COVID‐19 vaccines. Cell. 2022;185:2434‐2451.e2417. doi:10.1016/j.cell.2022.05.022 35764089 PMC9135677

[98] Wang F , Zuroske T , Watts JK . RNA therapeutics on the rise. Nat Rev Drug Discov. 2020;19:441‐442. doi:10.1038/d41573-020-00078-0 32341501

[99] Ou BS , Saouaf OM , Baillet J , Appel EA . Sustained delivery approaches to improving adaptive immune responses. Adv Drug Deliv Rev. 2022;187:114401. doi:10.1016/j.addr.2022.114401 35750115

[100] Damase TR , Sukhovershin R , Boada C , Taraballi F , Pettigrew RI , Cooke JP . The limitless future of RNA Therapeutics. Front Bioeng Biotechnol. 2021;9:628137. doi:10.3389/fbioe.2021.628137 33816449 PMC8012680

[101] Hou X , Zaks T , Langer R , Dong Y . Lipid nanoparticles for mRNA delivery. Nat Rev Mater. 2021;6:1078‐1094. doi:10.1038/s41578-021-00358-0 34394960 PMC8353930

[102] Truong B , Allegri G , Liu XB , et al. Lipid nanoparticle‐targeted mRNA therapy as a treatment for the inherited metabolic liver disorder arginase deficiency. Proc Natl Acad Sci USA. 2019;116:21150‐21159. doi:10.1073/pnas.1906182116 31501335 PMC6800360

[103] Gurung S , Timmermand OV , Perocheau D , et al. mRNA therapy restores ureagenesis and corrects glutathione metabolism in argininosuccinic aciduria. bioRxiv. 2022. doi:10.1101/2022.10.19.512931 PMC7615535 38198573

[104] Arcturus Therapeutics I . Phase 1b study to assess safety, tolerability, and pharmacokinetics of ARCT‐810 in stable adult subjects with ornithine transcarbamylase deficiency . https://www.clinicaltrials.gov/ct2/show/NCT04442347 2020.

